# Combining NeuroPainting with transcriptomics reveals cell-type-specific morphological and molecular signatures of the 22q11.2 deletion

**DOI:** 10.1101/2024.11.16.623947

**Published:** 2024-11-17

**Authors:** Matthew Tegtmeyer, Dhara Liyanage, Yu Han, Kathryn B. Hebert, Ruifan Pei, Gregory P. Way, Pearl V. Ryder, Derek Hawes, Callum Tromans-Coia, Beth A. Cimini, Anne E. Carpenter, Shantanu Singh, Ralda Nehme

## Abstract

Neuropsychiatric conditions pose substantial challenges for therapeutic development due to their complex and poorly understood underlying mechanisms. High-throughput, unbiased phenotypic assays present a promising path for advancing therapeutic discovery, especially within disease-relevant neural tissues. Here, we introduce NeuroPainting, a novel adaptation of the Cell Painting assay, optimized for high-dimensional morphological phenotyping of neural cell types, including neurons, neuronal progenitor cells, and astrocytes derived from human stem cells. Using NeuroPainting, we quantified cell structure and organelle behavior across various brain cell types, creating a public dataset of over 4,000 cellular traits. This extensive dataset not only sets a new benchmark for phenotypic screening in neuropsychiatric research but also serves as a gold standard for the research community, enabling comparisons and validation of results. We then applied NeuroPainting to identify morphological signatures associated with the 22q11.2 deletion, a major genetic risk factor for schizophrenia. We observed profound cell-type-specific effects of the 22q11.2 deletion, with significant alterations in mitochondrial structure, endoplasmic reticulum organization, and cytoskeletal dynamics, particularly in astrocytes. Transcriptomic analysis revealed reduced expression of cell adhesion genes in 22q11.2 deletion astrocytes, consistent with recent post-mortem findings. Integrating the RNA sequencing data and morphological profiles uncovered a novel biological link between altered expression of specific cell adhesion molecules and observed changes in mitochondrial morphology in 22q11.2 deletion astrocytes. These findings underscore the power of combined phenomic and transcriptomic analyses to reveal mechanistic insights associated with human genetic variants of neuropsychiatric conditions.

## Full Text Availability

The license terms selected by the author(s) for this preprint version do not permit archiving in PMC. The full text is available from the preprint server.

